# Computing travel time when the exact address is unknown: a comparison of point and polygon ZIP code approximation methods

**DOI:** 10.1186/1476-072X-8-23

**Published:** 2009-04-29

**Authors:** Ethan M Berke, Xun Shi

**Affiliations:** 1 Department of Community and Family Medicine, Dartmouth Medical School, Hanover, New Hampshire, USA; 2 The Dartmouth Institute for Health Policy and Clinical Practice, Dartmouth Medical School, Hanover, New Hampshire, USA; 3 The Norris Cotton Cancer Center, Dartmouth Hitchcock Medical Center, Lebanon, New Hampshire, USA; 4 Veterans' Rural Health Research Center–Eastern Region, VA Medical Center, White River Junction, Vermont, USA; 5 Department of Geography, Dartmouth College, Hanover, New Hampshire, USA

## Abstract

**Background:**

Travel time is an important metric of geographic access to health care. We compared strategies of estimating travel times when only subject ZIP code data were available.

**Results:**

Using simulated data from New Hampshire and Arizona, we estimated travel times to nearest cancer centers by using: 1) geometric centroid of ZIP code polygons as origins, 2) population centroids as origin, 3) service area rings around each cancer center, assigning subjects to rings by assuming they are evenly distributed within their ZIP code, 4) service area rings around each center, assuming the subjects follow the population distribution within the ZIP code. We used travel times based on street addresses as true values to validate estimates. Population-based methods have smaller errors than geometry-based methods. Within categories (geometry or population), centroid and service area methods have similar errors. Errors are smaller in urban areas than in rural areas.

**Conclusion:**

Population-based methods are superior to the geometry-based methods, with the population centroid method appearing to be the best choice for estimating travel time. Estimates in rural areas are less reliable.

## Background

Spatial accessibility is an important factor in assessing overall access to healthcare, and road-network-based travel time has become a popular way to measure this component of accessibility. As geographic information systems (GIS) become increasingly available, public health practitioners and researchers now have easy-to-use tools to calculate travel times that once were technically and computationally beyond the reach of most, especially for large datasets. A challenge remains, however, in how to estimate subjects' travel times when the exact address is not available. Due to confidentiality and/or data quality reasons, point location information of subjects is often aggregated to larger areal units. The ZIP code is often the finest granularity of geography available to health researchers, and studies of healthcare accessibility or distance are commonly based on travel times estimated from a subject's ZIP code to a known destination. While ZIP codes are actually collection of postal delivery routes that are modifiable at the level of the postmaster, they are still a commonly used geographic unit in health research. An important question that arises is how to measure a distance from a polygon (i.e. ZIP code area) to a point (i.e. exact, known address of a destination or a facility). Different strategies for handling this issue may result in varying outputs, which may in turn lead to different conclusions about accessibility. We are not aware of any formal comparison and validation of different methods in the public health literature.

Practical methods of estimating travel time from aggregate data can be based on point model or polygon models. The point model assumes that all subjects within an areal unit are concentrated on a single point, whereas the polygon model assumes that the subjects are spread across the unit. Both models can take either a geometric or population-based approach: (1) a geometric point method uses the geometric centroid of an areal unit as the origin for all the subjects from that unit when calculating travel times [1,2]; (2) a population-based point method uses the population centroid of an areal unit as the origins for the subjects [3]; (3) a geometric polygon method creates travel time zones around facilities and assigns subjects assuming an even distribution across an aggregate unit [4,5]; (4) a population-based polygon method assigns subjects to a travel time zones under the assumption that the locations of the subjects within a unit follows the distribution of the population in that unit.

In this study, we sought to determine the best of the above four methods by comparing their outputs with actual travel times calculated from subjects' exact locations.

## Methods

### Data sources

We first identified two states to compare different methods of address estimation. New Hampshire and Arizona demonstrate a number of important characteristics that allow for our results to be more generalizable to the rest of the nation. New Hampshire is a small New England state of 24,239 km^2 ^and a population of 1,317,987 [6]. The state is predominately rural, with only one population center of over 100,000 people (Manchester). The mean population density is 110 people per km^2^, though the median is 33. Arizona is comparably a larger state of 295,254 km^2 ^and a population of 5,882,273 [6]. The mean population density is 608 people per km^2 ^though the median is 31.8. There are multiple population centers, including the large cities of Phoenix and Tucson.

For each state, we obtained residential, geocoded addresses from a national data firm (Melissa DATA Corporation, Rancho Santa Margarita, California). Nationally, the database contains 142 million total records and is updated monthly. In NH, there were 614,211 possible addresses, and in AZ there were 2,872,268 possible addresses. Our initial sampling strategy was to make the spatial distribution of subjects follow the distribution of the population, which mimics the distribution of actual subjects. However, if we apply a single sampling scheme to the entire state, most samples will be concentrated in urban areas and many rural areas will not be represented. We chose the ZIP code as our aggregate unit of analysis due to its popularity and availability in health research. We hypothesized distance estimates in urban and rural areas might vary, as ZIP code areas are usually smaller in urban than rural areas. Based on this assumption, we requested separate sampling processes in urban and rural areas for both states. Acknowledging that different rural classification systems my categorize some areas differently and that there is no "right" system of classification [7], we chose to use the urban-rural scheme provided by ESRI for consistency with the other available sources of data. For New Hampshire, we requested a total of 2,000 samples, with 100 sampled from urban areas. For Arizona, we requested a total of 3,000 samples, with 400 sampled from urban areas. We received 1,998 samples for New Hampshire, with 101 in urban areas, and 3,016 samples for Arizona, with 413 in urban areas. Data were selected for each town, stratified as urban or rural. The number of samples allocated to each town was proportional to the total number of address records available for that town, which was correlated to the population size of the town. Every 10^th ^address record was picked until the required number of samples were met. For rural towns with very small populations, we oversampled to ensure every town had at least one subject. The data company geocoded all the selected subjects to the longitude and latitude at a precision of 6 digits to the right of the decimal. We associated each geocoded subject to their ZIP code area.

Destinations were designated as the geocoded address of each state's National Cancer Institute Comprehensive Cancer Center or affiliated satellites [8]. The locations were determined through geocoding the official addresses of each center or satellite from the cancer center's web site. There was 1 destination associated with the Cancer Center in Arizona, and 3 in New Hampshire (1 of the 3 was located in Vermont, immediately across the border from New Hampshire).

The data of ZIP code areas, urban areas, road network, and Census Block centroids and their associated population were from data included in our GIS package (ArcGIS, ESRI, Redlands, CA). Source data for ZIP Code areas and Census Blocks were from TeleAtlas North America and ESRI, with source data for Urban Areas from the US Census and ESRI. The calculation of travel time was based on the 2006 ESRI street network dataset. These data are based on U.S. Census TIGER line data for street centerlines, with Census Feature Class Codes (CFCC) assigned to each line segment to provide speed limit information. CHCC codes classify road types and assign a corresponding speed limit (i.e. CFCC code 'A00' is classed as Road: Major and minor categories unknown and given the speed limit 40 mph). LandScan Global population data, for characterizing the spatial distribution of population within a ZIP code area, were obtained from the Oak Ridge National Laboratory, described below [9]. All data were transformed to their corresponding State Plane Coordinate Systems to minimize errors in distance and area calculations.

### Actual Travel Time

**Figure 1:**
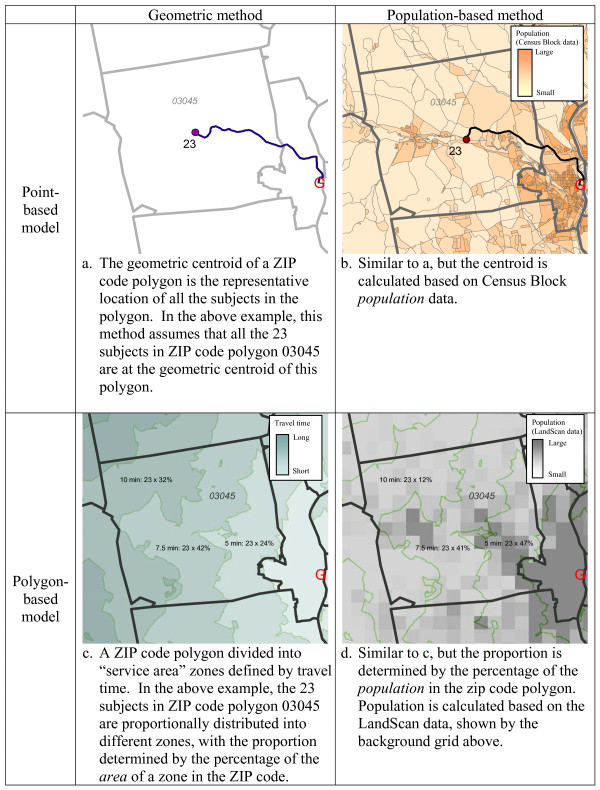
**Examples of 4 methods of approximating distance to a facility from an estimated address**.

Use of roads by motorized vehicles is often the preferred method of travel to reach health facilities in developed countries [10]. We therefore calculated travel time based on the travel distance over the road network from an origin to a destination and speed limits for each segment of road using Network Analyst in ArcGIS. Details of the implementations of the four methods of approximation are as follows and illustrated in Figure 1.

### Geometric point method

For each ZIP code area, the geometric centroid (the point at the geometric center of the area) was used as the common origin of all the subjects falling into that ZIP code. The assumption of this method was that all the subjects live at the geometric center of the area, and consequently all have the same travel time and use the same nearest facility.

### Population-based point method

We located the population centroid of each ZIP code area through weighted averaging of the geometric centroids of all the Census Blocks within the ZIP code, with the population of the Census Blocks used as the weights. We used this weighted population centroid as the common origin of all the subjects falling into the ZIP code.

### Geometric polygon method

The implementation of a polygon method is different from that of the point methods in that instead of measuring travel time from point to point, it defines travel time zone(s) around a facility. Subjects' travel time is based on which time ring falls on their location. In our study, we created travel time zones using the service area function of Network Analyst in ArcGIS. This function created a buffer area around a specified facility based on travel time (e.g. a 30-minute service area of a hospital defines the area where subjects can reach the hospital within 30 minutes). We created a series of service area rings (i.e. travel time zones) around each of the cancer centers or satellites, using 5-minute intervals, until the entire state had coverage. When travel time zones of different facilities overlapped each other, we preserved the one with the shortest travel time. Once travel time zones were defined, subjects were allocated to the zones to obtain travel time estimates. Under the geometric approach, the number of subjects from a ZIP code area allocated to a specific travel time zone was based on the proportion of the ZIP code area falling into the zone (e.g. if a ZIP code area has 10 subjects and 30% of the ZIP code (in terms of area) fell into the travel time zone of 10–15 minutes, then 3 subjects would be allocated to that zone). This geometric approach only considers the geometry of the ZIP code (shape and area) and assumes that the subjects are distributed evenly across the area.

### Population-based polygon method

The difference between this method and the geometric polygon method only lies in how to allocate subjects in each ZIP code area into travel time zones. Instead of assuming that the subjects are evenly distributed across a ZIP code area, this method allocates subjects under the assumption that the subjects follow the distribution of population. This method requires spatially detailed information about population distribution within a ZIP code. We used LandScan Globe data for this purpose, with a resolution in New Hampshire and Arizona of approximately 800 m × 800 m, which is smaller than the sizes of most ZIP code areas in the two states. LandScan data, from the Oak Ridge National Laboratory, allocates U.S. Census counts of the population to equally-sized cells. The value of each 800 m × 800 m cell is the total number of people within the area represented by that cell based on likelihood coefficients derived from imaging data of land cover, road proximity, lighted areas at night, and slope of the land [9]. Land cover and lighted areas at night are collected through remote sensing, and models are verified with high-resolution images. We first allocated all the subjects in a ZIP code into the LandScan cells enclosed by the ZIP code area. The number of subjects in each cell was determined by the proportion of the people in that cell from the total population of the ZIP code. Using the spatial location of each cell, we identified the corresponding travel time zone.

### Validation

We used travel times calculated based on the individual exact locations as the "true value" to validate the estimates from the four methods presented above. We computed the mean absolute error (MAE, calculated as , where *t*_*i *_is the travel time calculated based on individual exact location,  is the estimated travel time based on aggregate data, and *n *is total number of subjects), the root mean square error (RMSE, calculated as ), and Pearson's correlation coefficient. A larger MAE or RMSE indicate a less accurate estimation. We performed paired t-tests to determine whether the estimates were significantly different from the true values and if the results from different methods were significantly different from each other.

We also investigated the relationship between the accuracy of the estimate and the size of the ZIP code polygon, and that between the accuracy and the population density of the polygon. Specifically, we first calculated the MAE and MSE of each method for each polygon, then calculated the correlation coefficients between these error measurements and the sizes and population densities of the polygons.

## Results

Of the 242 ZIP codes in New Hampshire, the size ranged from 1.6 to 958 square kilometers (mean 98). In Arizona, the 319 ZIP codes ranged from 0.4 to 10,057 square kilometers (mean 925). The overall mean travel time from individual residence to cancer facility in Arizona was 185 minutes (range: 0.84 – 719 minutes). In urban areas, travel times were markedly less (98.1 minutes, range: 0.84 – 160 minutes), while in more rural areas travel times were slightly above the average (197 minutes, range: 21 – 719 minutes). The median travel time was 169 minutes overall, 115 minutes in urban areas, and 214 minutes in rural areas. In New Hampshire, the overall mean travel time from individual residence to cancer facility was 41 minutes (range: 0 – 159 minutes). In urban areas, travel times were again shorter (12.6 minutes, range: 0.74 – 24 minutes), while in more rural areas travel times were longer (42.6 minutes, range: 0 – 159 minutes), though there was less of a range than seen in Arizona. The median travel time was 36.8 minutes overall, 14.7 minutes in urban areas, and 37.7 minutes in rural areas.

The MAE and RMSE values (Table 1) for both states show that 1) the population-based methods had smaller errors than the geometric methods; 2) within the same category (geometry- or population-based), the point method and the polygon method had similar accuracy; 3) except for the population-based point method, the other three methods tended to overestimate the travel time (in rural areas of Arizona, overall the two geometric methods overestimated by nearly 20 minutes); 4) the estimate errors in rural areas were always greater than their counterparts in urban areas; and 5) the errors in New Hampshire were generally smaller than the errors in Arizona. In terms of the magnitude of error, the contrast between urban and rural areas was less striking in New Hampshire than in Arizona. Additionally, performance of the point methods and polygon methods was not consistent across states or across urban and rural areas. For example, in Arizona the population-based point method was slightly less accurate than the population-based polygon method in urban areas (2.3 vs. 2.1 for MAE and 4.5 vs. 3.4 for RMSE) but was more accurate (in terms of RMSE) in rural areas; in New Hampshire, however, the pattern was reversed.

**Table 1:** Variation in estimated travel time compared to the true travel time for Arizona and New Hampshire.

Method	OVERALL			URBAN			RURAL		
	
	AVERAGE TIME (min)	MAE^1^	RMSE^2^	AVERAGE TIME (min)	MAE^1^	RMSE^2^	AVERAGE TIME (min)	MAE^1^	RMSE^2^
ARIZONA									

Actual Time	185			98.1			197.4		

Population Centroid	185.8	5.2	8.9	97.5	2.3	4.5	198.4	5.6	9.3

Geometric Centroid	201.4	21.1	34.4	99.3	3.5	7.1	215.9	23.6	36.7

Service area based on LandScan	187.8	5.4	13.2	98.5	2.1	3.4	200.6	5.8	14.1

Service area based on area	202.5	20.7	34.7	100.9	4.0	9.3	216.9	23.1	37.0

NEW HAMPSHIRE									

Actual Time	41.1			12.6			42.6		

Population Centroid	40.7	2.3	12.0	12.6	1.5	4	42.2	2.4	12.5

Geometric Centroid	41.9	3.0	18.1	13.7	2.1	13.1	43.5	3.0	18.4

Service area based on LandScan	41.6	2.0	11.5	12.8	1.5	4.3	43.1	2.0	11.9

Service area based on area	43.3	3.1	27.8	14.4	2.5	14.2	44.9	3.1	28.5

(1) MAE: Mean Absolute Error; (2) RMSE: Root mean square error

All methods demonstrated correlation coefficients greater than 0.9 when compared to the actual address in Arizona (Table 2). The geometric centroid method had the lowest correlation in urban areas at 0.96. Correlation coefficients in New Hampshire were similar. Pairwise t-tests demonstrated that estimation methods only tended to agree with each other (as opposed to the true address as measured by the correlation coefficient) in urban areas of New Hampshire where ZIP codes are generally of smaller size, though even in this sub-population the agreement was not uniform (data not shown). In the majority of comparisons for urban, rural, and overall groupings, however, mean estimates of time were statistically different from each other.

**Table 2:** Pairwise correlations comparing actual travel times to four estimation methods, for urban and rural areas of Arizona (AZ) and New Hampshire (NH).

	Estimation Method			
	
Location of actual travel time	Geometric Centroid	Population Centroid	Service area based on LandScan	Service area based on area
AZ Urban	0.957*	0.994*	0.991*	0.957*

AZ Rural	0.988*	0.995*	0.997**	0.980*

NH Urban	0.920**	0.966	0.967	0.933*

NH Rural	0.988*	0.991*	0.992*	0.986*

* p < 0.001

** p < 0.05

† NS

Correlation coefficient values demonstrated fairly strong positive relationships between the estimated travel time error and the size of polygon (i.e. larger polygons tended to have greater errors) (Table 3). The relationship between the error and population density was less strong, but was consistently negative (i.e., the lower the population density, the greater the error). The two population-based methods were less sensitive to the size and population density of the polygon than the two geometry-based methods. The population-based point method was the least sensitive one among all the methods.

**Table 3:** Pairwise Correlation between MAE^1 ^and RMSE^2 ^and ZIP code area and population density for Arizona and New Hampshire.

	Estimation Method			
	
Error Measurement Method	Geometric Centroid	Population Centroid	Service area based on LandScan	Service area based on area
ARIZONA				

MAE – area	0.535*	0.410*	0.417*	0.578*

RMSE – area	0.489*	0.221*	0.315*	0.330*

MAE – population density	-0.357*	-0.244*	-0.271*	-0.432*

RMSE – population density	-0.207*	-0.1143**	-0.148*	-0.196**

NEW HAMPSHIRE				

MAE – area	0.552*	0.302*	0.274*	0.552*

RMSE – area	0.517*	0.173**	0.256*	0.476*

MAE – population density	-0.202**	-0.160**	-0.189**	-0.220**

RMSE – population density	-0.136**	-0.074	-0.104	-0.139**

(1) MAE: Mean Absolute Error; (2) RMSE: Root mean square error

* p < 0.001

** p < 0.05

† NS

## Discussion

In this study we quantitatively compared four different methods of estimating travel time when the subject's location data were only available at the area unit level. We summarize our findings from this study as follows: 1) the estimates from all the methods were statistically different from the travel times calculated based on the actual addresses; 2) in terms of accuracy, the two population-based methods were superior to the two geometric methods; 3) within the same category (geometric or population-based), the overall accuracies of the point method and the polygon method were not considerably different, though the point method seemed to be more accurate than the polygon method; 4) the larger the areal unit or the lower the population density, the less accurate the estimate, with accuracy having a stronger relationship to the size of the areal unit than the population density; 5) estimates in rural areas were less reliable than those in urban areas, calling for special attention to those states with large rural areas and/or states with both large metropolitan areas and rural areas (e.g. Arizona) when estimating travel time using polygon-level data and drawing conclusions based on summarized statistics.

Based on our results, we recommend the population-based point method as the best choice for the following reasons: 1) in our testing, it was the most accurate method; 2) this method was least sensitive to the size of the areal unit and population density; 3) Census Block data for calculating the population centroid are readily available; and 4) the process of calculating the population centroid is computationally easier than the process of first calculating service area rings and then allocating subjects based on population distribution.

There were relative high correlations among all methods. This may indicate that the general trends in the estimates from different methods are very similar leading to a conclusion that if we were interested in the relative difference in travel times (e.g., in a social justice study), analyses using different estimation methods may give similar results. On the other hand, if we are interested in the absolute difference in travel times (e.g. in a planning project or positioning a new health facility), the accuracy of the estimated travel time might be more critical and the difference among different methods might be important.

We were surprised by the fact that the computationally difficult population-based polygon method using the LandScan data was not superior to other methods. In terms of overall accuracy, it was not substantially different from the recommended population-based point method. We suspect two reasons for this observation: 1) in urban areas, the resolution of the LandScan data (approximately 800 m × 800 m) is much lower than that of the Census Block data used by the point method; and 2) in rural areas, where the LandScan data is more precise than the Census Block data, the number of subjects in a ZIP code polygon is usually small and may have been better "captured" by the "concentrating" strategy of the point method than by the "spreading" strategy of the polygon method. Nevertheless, we believe that the population-based polygon method is potentially promising, as it has the capacity of incorporating more spatially-detailed population data when they are available.

Our analysis showed that choosing a travel time method based on the geometric centroid method may lead to greater errors in estimation. Using the geometric center of a ZIP code without taking into consideration the distribution of population led to considerable bias, particularly in rural areas with larger ZIP codes. In regions with shorter travel times to destinations, the choice of estimation method is less important, though population-based methods still appear to be superior. The variation across methods in NH was less than that of AZ. This may be due to the fact that travel times in NH are much shorter than those of AZ, and as a result the absolute differences across different methods are expectedly smaller in NH than in AZ. The travel times of NH are smaller because NH is a much smaller state, and its patients are closer to multiple cancer centers, whereas AZ only has one cancer center. In addition the ZIP code polygons in NH are relatively smaller compared with some large polygons in AZ. With small polygons, the absolute difference between methods, e.g., the distance between the geometric centroid and population centroid, will be less drastic.

The literature on geographic accessibility dates back over 30 years [11,12], and some observations made then about potential errors in assessment of time or distance using proxy measures of address are still important to note. Hillsman and Rhoda describe a number of potential errors when determining distance, or as an extension, time, to a destination. The issue of multiple service destinations may create error by estimating the distance to the wrong service center (described as Source C error) [13]. Using the geometric as opposed to the population-based centroid may exacerbate this area in situations of multiple destinations, such as New Hampshire in our simulation. It is also possible that the distance to a service center within the same aggregate unit as the origin address may result in distances that underestimate the actual location (notes as Source B error by Hillsman et al.). By using a population-based point method, we hope to mitigate this error by not assuming equal dispersion of the population throughout the polygon. Improved network routing algorithms in modern GIS software should help to mitigate Source C errors. Another issue well-described in the literature that deserves mention is the choice of aggregation level. Francis et al. describe the balance between reducing aggregation error by using smaller units of aggregation and computational effort and data availability[14]. The ratio of subject locations (demand points) to facilities is crucial to consider; as the number of subject locations increases with a fix number of facilities, the error decreases. As this ratio decreases, the risk of error may increase, and users should consider increasing the number of aggregated spatial units or redefine spatial zoning (while considering the effect of MAUP) to mitigate errors.

Our findings build on the work of others studying geographic accessibility and methods of aggregation. Apparicio, et al. examined population-weighted and geographic centroids and found a 5 to 10% difference in measurement between them in urban areas [15]. This is in alignment with our findings, which we expand to include rural areas as well as estimation methods based on polygon approaches. Our work adds to the growing body of literature on understanding the dynamics between demand for services and locations of subjects. Health services research that uses distance measures, including the development of Primary Care Service Areas to evaluate resource utilization among primary care practices, will benefit from refined methods to approximate patient addresses [16]. Researchers using the floating catchment method [17] will also be better informed by the current study when choosing an appropriate strategy to define the catchment of a health care facility. As noted by Rushton [18], appropriate geocoding of the population and facilities is necessary for this method to work, and an improved method of estimating an actual address from the ZIP code should improve the results of this method.

Our methods have a number of limitations that warrant further research. We were not able to account for traffic, road conditions, or weather in our network distance analysis, and were unable to test if there was a significant difference in these factors between Arizona and New Hampshire. Similarly, we did not test all possible travel time bands for the LandScan or area-based travel ring method – we instead choose a granularity deemed fine-grained to account for clinically relevant travel variability in urban areas that is computationally efficient.

It is possible that our actual addresses had some error in geocoding or placement along a street network, though this would impact all methods of estimation equally. While we tried to include a nationally representative sample by choosing two states of different size and location, it is possible that other states or nations may exhibit different road network characteristics that could potentially impact our conclusions.

Our methods of distance estimation relied on a network analysis based on available road data. We did not consider travel off the street network as a way to reach a destination in this discussion. As noted previously, most patients in the U.S. and other developed countries will utilize the street network to reach a health facility, though in developing countries this may not be the case [19]. In these situations, a raster-based model using a least-cost path approach may be the preferred method of distance estimation [20]. These methods do not limit travel across a road network as network methods require, but instead allow travel across terrain, and can account for barriers such as rivers [21]. These methods require considerably more data depending on the complexity of the least-cost model, including land use, elevation, road data, and barriers. While we believe a network analysis utilizing posted road speeds is a more accessible approach to most users and provides an extremely accurate measure of travel time, researchers studying travel time across terrain without streets should consider this alternate method of analysis.

## Conclusion

Despite these limitations, we believe that the best method of distance estimation need not be the most complicated. Population-based methods are superior to the geometry-based methods, with the population centroid method appearing to be the best choice for estimating travel time. Appropriate methods of estimation are particularly important in rural areas, and in areas of low population density. Researchers should thoroughly examine their geographic data to determine which method of estimation is most reasonable.

## Competing interests

The authors declare that they have no competing interests.

## Authors' contributions

EB and XS contributed equally to the concept, design, data acquisition, analysis, and interpretation of results. Both authors wrote and approved the final manuscript.
